# Associations Between Polypharmacy and Cognitive and Physical Capability: A British Birth Cohort Study

**DOI:** 10.1111/jgs.15317

**Published:** 2018-03-24

**Authors:** Mark James Rawle, Rachel Cooper, Diana Kuh, Marcus Richards

**Affiliations:** ^1^ Unit for Lifelong Health and Ageing, Medical Research Council University College London London United Kingdom

**Keywords:** polypharmacy, cognition, physical capability, longitudinal, life‐course

## Abstract

**Objectives:**

To investigate longitudinal associations between polypharmacy and cognitive and physical capability and to determine whether these associations differ with cumulative exposure to polypharmacy.

**Design:**

Prospective birth cohort study.

**Setting:**

England, Scotland, and Wales.

**Participants:**

An eligible sample of men and women from the Medical Research Council National Survey of Health and Development with medication data at age 69 (N=2,122, 79%).

**Measurements:**

Cognitive capability was assessed using a word learning test, visual search speed task, and the Addenbrooke's Cognitive Examination, Third Edition (ACE‐III). Physical capability was measured using chair rise speed, standing balance time, walking speed, and grip strength.

**Results:**

Polypharmacy (5–8 prescribed medications) was present in 18.2% of participants at age 69 and excessive polypharmacy (≥9 prescribed medications) in 4.7%. Both were associated with poorer cognitive and physical capability in models adjusted for sex, education, and disease burden. Stronger associations were found for excessive polypharmacy (e.g., difference in mean ACE‐III scores comparing polypharmacy=−2.0, 95% CI=−2.8 to −1.1 and excessive polypharmacy=−2.9, 95% CI=−4.4 to −1.4 with no polypharmacy). Participants with polypharmacy at age 60 to 64 and at age 69 showed stronger Negative associations with cognitive and physical capability were stronger still in participants with polypharmacy at both age 60 to 64 and at age 69 (e.g. difference in mean chair rise speed, comparing polypharmacy with no polypharmacy at both ages=−3.9, 95% CI=−5.2 to −2.6 and at age 60–64 only=−2.5, 95% CI=−4.1 to −0.9).

**Conclusion:**

Polypharmacy at age 60 to 64 and age 69 was associated with poorer physical and cognitive capability, even after adjusting for disease burden. Stronger negative associations were seen in participants with longstanding polypharmacy, suggesting a cumulative, dose‐dependent relationship (where dose is the number of prescribed medications). Future research aiming to improve cognitive and physical capability should consider interventions to reduce the duration and level of polypharmacy at younger ages, in addition to optimizing disease control with appropriate medications.

Polypharmacy is a growing phenomenon in the United Kingdom, with a little more than one‐fifth of the adult population now prescribed more than 5 medications.^1^ Particularly at risk are older adults, individuals with lower levels of education, and those with higher levels of disease burden.^2^ Polypharmacy itself is associated with numerous negative clinical outcomes, including greater risk of falls, premature mortality, and adverse drug reactions.^3^ Associations between polypharmacy and objective measures of physical impairment (in particular lower limb function) have been noted in observational cohort studies,^4^, ^5^ suggesting that polypharmacy may have an effect on underlying physical capability, leading to these negative clinical outcomes. In a prospective cohort study of 294 individuals aged 75 and older, individuals taking more than 10 medications were less able to perform instrumental activities of daily living and had lower Mini‐Mental State Examination (MMSE) scores, even when accounting for disease burden,^6^ than those taking fewer than 10 medications.

Despite these findings, trials studying the effect of medication reduction on clinical measures of cognitive and physical capability have found no associated improvements after medication cessation.^7^, ^8^, ^9^, ^10^ There are at least 2 possible unexplored reasons for this observed lack of effect. The first is that studies have focused on broad outcome measures, such as fewer falls, rather than subtler changes in physical and cognitive capability. The second is that prolonged rather than contemporaneous polypharmacy may have a stronger influence on physical and cognitive capability.

To address these important gaps, we examined associations between polypharmacy and detailed measures of physical and cognitive capability in a population‐representative, age‐homogenous birth cohort, adjusting for disease burden. We hypothesized that higher levels of polypharmacy would be associated with poorer cognitive and physical capability and that these associations would be more pronounced with longer exposure to polypharmacy.

## Methods

The Medical Research Council National Survey of Health and Development (NSHD), has followed 5,362 individuals (2,547 female) since their birth in England, Scotland, or Wales in a single week of March 1946, so far to age 71.^11^, ^12^ The most recent data collection was conducted when participants were aged 68 to 69. After responding to a postal questionnaire, participants still alive and with a known current address in mainland Britain (n=2,698) were invited to have a home visit at age 69; 2,149 (79.7%) visits were completed. Invitations were not sent to those who had died (n=995), were living abroad (n=583), restricted participation to postal questionnaires (n=22), had previously withdrawn from the study (n=632), or had been lost to follow‐up (n=432).^11^

### Cognitive Capability Outcomes

Trained research nurses tested cognitive capability at age 69. Verbal memory was assessed using a 3‐trial 15‐item word learning task (range 0–45), with 2 word lists alternated over waves to minimize practice effects, and processing speed was assessed using a verbal search speed task, in which participants are asked to cross out randomly distributed letters ‘P’ and ‘W’ in a grid of other letters as quickly and accurately as possible in 1 minute (range 0–600). The Addenbrooke's Cognitive Examination, Third Edition (ACE‐III) was also administered; this is a cognitive screening battery designed to detect risk of Alzheimer's disease and cognitive impairment that is commonly used in clinical practice.^13^ It is scored across varying cognitive subdomains, with a maximum score of 100. Because verbal fluency is included, distribution of the total score is quasi‐normal and avoids the pronounced ceiling effect of most cognitive state tests.

### Physical Capability Outcomes

The research nurses administered 4 tests of physical capability at age 69 following standard protocols.^14^ To assess chair rise speed (number of stands/min), participants were timed standing up and sitting back down from a chair 10 times as fast as possible (or 5 times if they were unable to complete 10 rises (n=3)). Usual walking speed was recorded twice over a distance of 2.44 m from a standing start, with the faster of the 2 speeds used in analyses. Standing balance was measured as the length of time participants were able to stand on 1 leg with their eyes closed for a maximum of 30 seconds. A natural log‐plus‐1 transformation was used to take account of the skewed distribution of balance times. Grip strength was assessed using a Jamar electronic dynamometer in a seated position.^15^ Two measures per hand were recorded, with the maximum of all 4 measures achieved used in analyses. For all 4 variables, participants unable to complete the tests for health reasons (n=99 for chair rise speed, n=34 for maximum walk speed, n=111 for standing balance, n=24 for grip strength) were assigned a score equal to the mean of the sex‐specific lowest fifth for each measure, consistent with prior work in the NSHD.^16^

### Ascertainment of Polypharmacy

Research nurses collected information on regularly prescribed medication at age 69 and at the previous data collection at age 60 to 64.^12^ During both assessments, nurses recorded all regularly prescribed medications, including as‐needed medications that were regularly used, preferably using written lists that participants provided rather than relying on recall. If data were missing from the nurse interview at age 60 to 64, the same information collected in a postal questionnaire at the same age was substituted (n=62). From these data, we derived a total count of medications at both time points and an indicator of general polypharmacy adapted from preexisting thresholds,^17^ namely 5 to 8 medications (polypharmacy) and 9 or more medications (excessive polypharmacy). For analysis of longitudinal data, a 4‐category variable was derived that indicated whether polypharmacy (≥5 medications) was present at: neither age, 60 to 64 only, 69 only, or both ages.

### Covariables

Covariables were factors known to influence the risk of polypharmacy: sex, education, and disease burden.^2^, ^18^ In models of physical capability, we also included body mass index (BMI) and standing height, given the important influence of body size on performance on these tests.^15^, ^19^

Education was defined as highest educational qualifications achieved by age 26, grouped into three categories (none, General Certificate of Secondary Education ordinary secondary level or their equivalents, and advanced secondary level or higher). Disease burden at age 69 was defined according to measures. The first was a count of 0, 1, 2, or 3 or more self‐reported doctor‐diagnosed chronic diseases or disorders over the last 10 years. Disease severity was assessed according to binary responses to the question, “Do you have any long‐term illness, health problem or disability that limits the activities or work you can do?” Trained nurses measured weight (kg) and standing height (meters) during the home visit at age 69, which were used to calculate BMI. If height was missing at age 69, height recorded at age 60 to 64 was substituted (n=29).

Finally, for longitudinal models, equivalent measures of cognitive and physical capability assessed at age 60 to 64 were used to take account of baseline levels of capability. These measures were assessed using similar methodology and protocols as at age 69, with the exception of ACE‐III, which was not tested at age 60 to 64.

### Ethics

Ethical approval for the NSHD data collection at age 68 to 69 was obtained from the Queen Square Research Ethics Committee (14/LO/1073) and the Scotland A Research Ethics Committee (14/SS/1009). At each stage of data collection, all participants provided written informed consent.

### Statistical Methods

Associations between polypharmacy and cognitive and physical capability were tested using linear regression models. Formal tests in initial models showed no evidence of an interaction with sex, so all subsequent models were sex‐adjusted. A stepwise process initially tested simple sex‐adjusted associations between exposure and outcome (Model 1), followed by adjustment for education and disease burden for all outcomes, with additional adjustment for BMI and standing height for physical capability outcomes (Model 2). For longitudinal associations, an additional model also adjusted for the equivalent cognitive or physical outcome measure at age 60 to 64 to estimate any association between polypharmacy and change in capability (Model 3). ACE‐III measures were omitted from Model 3 because no ACE‐III data were available for age 60 to 64. Sensitivity analyses were conducted on sex‐adjusted models on the maximum sample for each outcome measure and excluding those who were unable to complete physical capability tests for health reasons. Additional sensitivity analyses were conducted to further examine participants without polypharmacy, subdividing the group into those with no medications and those with 1 to 4 medications. All statistical analyses were conducted using Stata version 14 (Stata Corp, College Station, TX).

## Results

Of the 2,122 participants who had medication data at age 69, 2,121 (99.9%) had at least 1 measure of cognitive or physical capability. Of these participants, 2,007 (94.6%) had complete data for all covariables used in cognitive models and 1,989 (93.8%) for all covariables used in physical capability models. Medication data were also available at age 60 to 64 for 1,980 (93.4%) participants. Of these 1,980 participants who had medication data at age 60 to 64 and 69, 1,877 (94.8%) had complete data for all cognitive capability covariables, and 1,863 (94.0%) had complete data for all physical capability covariables. With regard to specific physical outcomes, 1,749 (93.9%) of these had data on chair rise speed, 1,759 (94.4%) on maximum walking speed, 1,833 (98.4%) on standing balance, and 1,855 (99.6%) on maximum grip strength. For cognitive outcomes, 1,719 (96.4%) had data on word learning task score, 1,746 (97.9%) on verbal search speed task score, and 1,445 (81.0%) on total ACE‐III score. Characteristics of the selected sample and those excluded for missing data are provided in Tables 1, 2, 3.

**Table 1:** Participant Characteristics for Total Cohort and Those Missing Data

Characteristic	Total Cohort, n = 2,007	Missing Data, n = 363^a^
Female, n (%)	1,027 (51.2)	170 (46.8)
Educational status, n (%)		
No formal education	626 (31.2)	104 (43.0)
Vocational, General Certificate of Secondary Education, or O‐level	567 (28.3)	65 (26.9)
≥A‐level	814 (40.6)	73 (30.2)
Number of doctor‐diagnosed diseases, n (%)		
0	493 (24.6)	72 (26.1)
1	693 (34.5)	80 (29.0)
2	409 (20.4)	46 (16.7)
≥3	412 (20.5)	78 (28.3)
Limiting disease, n (%)	539 (26.9)	107 (32.7)
Body mass index, kg/m^2^, mean ± SD	28.1 ± 5.2	28.9 ± 6.0
Height, m, mean ± SD	1.7 ± 0.1	1.7 ± 0.1

a 363 participants were interviewed at age 69 but were excluded from analysis because they were missing data for one or more covariables.

SD = standard deviation.

**Table 2:** Polypharmacy According to Age Group

	Total Cohort, n = 2,007		Missing Data, n = 363^a^	
	60–64	69	60–64	69
Polypharmacy (Medications, n)	n (%)			
No polypharmacy (≤4)^b^	1,517 (80.8)	1,547 (77.1)	167 (80.7)	183 (70.6)
Polypharmacy (5–8)	292 (15.6)	366 (18.2)	29 (14.0)	56 (21.6)
Excessive polypharmacy (≥9)	68 (3.6)	94 (4.7)	11 (5.3)	20 (7.7)

a 363 participants were interviewed at age 69 but were excluded from analysis because they were missing data for one or more covariables.

b Of the included participants without polypharmacy, 551 (29.1%) were prescribed no medications at age 60–64 and 395 (19.7%) at age 69. Of those missing, 63 (30.4%) were prescribed no medications at age 60–64 and 55 (21.2%) at age 69. The rest were prescribed 1–4 medications.

**Table 3:** Cognitive and Physical Capability According to Sex

	Total Cohort, n = 2,007		Missing Data, n = 363^a^	
	Male	Female	Male	Female
Capability at Age 69	Mean ± Standard Deviation			
Physical				
Chair rise speed, stands/min	27.0 ± 8.7	25.7 ± 8.5	26.7 ± 9.0	25.2 ± 8.4
Maximum walking speed, m/s	1.1 ± 0.3	1.0 ± 0.3	1.1 ± 0.3	1.0 ± 0.3
Standing balance time, log seconds	1.4 ± 0.6	1.3 ± 0.5	1.4 ± 0.6	1.3 ± 0.6
Maximum grip strength, kg	40.1 ± 8.4	23.9 ± 5.9	39.6 ± 9.5	23.5 ± 5.8
Cognitive				
Word‐learning task score	21.1 ± 6.0	23.2 ± 6.0	20.8 ± 5.6	22.4 ± 6.0
Verbal search speed task score	257.1 ± 75.1	268.0 ± 72.7	248.7 ± 67.7	263.9 ± 88.4
Addenbrooke's Cognitive Examination, Third Edition score	91.3 ± 5.9	91.6 ± 6.2	90.5 ± 5.4	91.7 ± 6.6

a 363 participants were interviewed at age 69 but were excluded from analysis because they were missing data for one or more covariables.

### Cross‐Sectional Associations Between Polypharmacy and Cognitive and Physical Capability at Age 69

**Figure 1:**
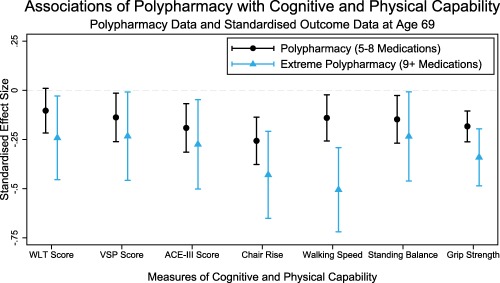
Standardized cross‐sectional associations between polypharmacy and cognitive and physical capability at age 69. All results adjusted for sex, education, and disease burden, plus body mass index and height in models of physical capability outcome (Model 2).

Three hundred sixty‐six (18.2%) participants had polypharmacy at age 69, and it was associated with poorer cognitive and physical capability on all measures, before and after covariable adjustment. For all outcomes, excessive polypharmacy, present in 94 (4.7%) participants, was more strongly associated with poorer performance than polypharmacy alone (Table 4). Of the cognitive measures, the largest effect sizes were seen for differences in mean ACE‐III score (polypharmacy: −2.0, 95% CI=−2.8 to −1.1; excessive polypharmacy; −2.9, 95% CI=−4.4 to −1.4, vs no polypharmacy). For physical capability, the largest effect sizes were seen for chair rise speed (polypharmacy: −2.2 stands/min, 95% CI=−3.2 to −1.2; excessive polypharmacy: −3.7 stands/min, 95% CI=−5.6 to −1.8, vs no polypharmacy). Standardized comparisons of these measures are shown in Figure 1 (data for figure provided in Supplementary Table S1).

**Table 4:** Cross‐Sectional Associations Between Polypharmacy and Cognitive and Physical Capability at Age 69

	Model 1		Model 2	
Outcome	Regression Coefficient^a^ (95% CI)	P‐Value	Regression Coefficient^a^ (95% CI)	P‐Value
Cognitive				
Word learning task, n = 1,934		<.001		.04
Polypharmacy	–1.7 (–2.3 to –1.0)		–0.6 (–1.3–0.1)	
Excessive polypharmacy	–3.2 (–4.5 to –1.9)		–1.5 (–2.8 to –0.2)	
Verbal search speed task, n = 1,964		<.001		.005
Polypharmacy	–17.1 (–25.6 to –8.7)		–12.5 (–21.6 to –3.4)	
Excessive polypharmacy	–27.6 (–43.0 to –12.1)		–20.8 (–37.5 to –4.2)	
Addenbrooke's Cognitive Examination, Third Edition, n = 1,673		<.001		<.001
Polypharmacy	–2.3 (–3.0 to –1.5)		–2.0 (–2.8 to –1.1)	
Excessive polypharmacy	–3.4 (–4.8 to –2.1)		–2.9 (–4.4 to –1.4)	
Physical				
Chair rise speed, stands/min, n = 1,864		<.001		<.001
Polypharmacy	–4.2 (–5.1 to –3.2)		–2.2 (–3.2 to –1.2)	
Excessive polypharmacy	–7.0 (–8.9 to –5.2)		–3.7 (–5.6 to –1.8)	
Walking speed, m/s, n = 1,876		<.001		<.001
Polypharmacy	–0.1 (–0.2 to –0.1)		0.0 (–0.1–0.0)	
Excessive polypharmacy	–0.3 (–0.4 to –0.2)		–0.2 (–0.2 to –0.1)	
Standing balance time, log seconds, n = 1,955		<.001		.02
Polypharmacy	–0.2 (–0.3 to –0.1)		–0.1 (–0.2–0.0)	
Excessive polypharmacy	–0.3 (–0.5 to –0.2)		–0.1 (–0.3–0.0)	
Grip strength, kg, n = 1,978		<.001		<.001
Polypharmacy	–3.0 (–3.8 to –2.2)		–2.0 (–2.8 to –1.1)	
Excessive polypharmacy	–5.4 (–6.9 to –3.8)		–3.7 (–5.3 to –2.1)	

No polypharmacy (reference) = 0–4 medications, polypharmacy = 5–8 medications, excessive polypharmacy = ≥9 medications.

Model 1: Adjusted for sex.

Model 2: Adjusted for sex, education and disease burden, plus body mass index and height in models of physical capability.

a Difference in mean score.

CI = confidence interval.

### Longitudinal Associations Between Polypharmacy and Cognitive and Physical Capability

**Figure 2:**
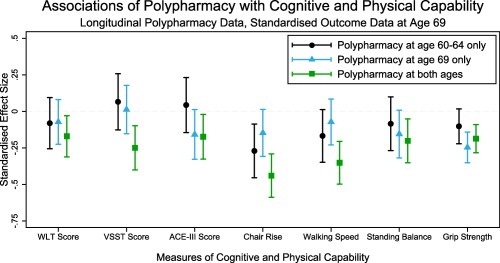
Standardized longitudinal associations between polypharmacy and cognitive and physical capability at age 69. All results adjusted for sex, education, and disease burden, plus body mass index and height in models of physical capability outcome (Model 2).

Participants with polypharmacy at both ages had lower mean cognitive and physical capability at age 69 than those without polypharmacy at either age, with the exception of grip strength (Table 5). The association between lower mean grip strength and polypharmacy was stronger at age 69 only (–2.6 kg, 95% CI=−3.8 to −1.3), although when additionally adjusting for grip strength at age 60 to 64, the strongest association was once again seen for those with polypharmacy at both ages (–1.9 kg, 95% CI=−2.9 to −0.9). Although participants with polypharmacy at just 1 age had better cognitive and physical capability than those with polypharmacy at both ages, they had poorer capability than those with polypharmacy at neither age (Figure 2, data for figure provided in Supplementary Table S2).

**Table 5:** Longitudinal Associations Between Polypharmacy and Cognitive and Physical Capability

	Model 1		Model 2		Model 3	
Outcome	Regression Coefficient^a^ (95% CI)	P‐Value	Regression Coefficient^a^ (95% CI)	P‐Value	Regression Coefficient^a^ (95% CI)	P‐Value
**Cognitive**						
Word learning task, n = 1,675		<.001		.22		.26
Polypharmacy at 60–64 only	–1.2 (–2.3–0.0)		–0.5 (–1.6–0.6)		–0.4 (–1.3–0.5)	
Polypharmacy at 69 only	–1.5 (–2.5 to –0.4)		–0.4 (–1.4–0.6)		–0.3 (–1.1–0.5)	
Polypharmacy at both ages	–2.1 (–3.0 to –1.3)		–0.9 (–1.8–0.0)		–0.7 (–1.4–0.0)	
Verbal search speed task, n = 1,705		<.001		.007		.04
Polypharmacy at 60–64 only	1.0 (–13.3–15.4)		5.7 (–8.8–20.1)		7.9 (–4.0 to –19.7)	
Polypharmacy at 69 only	–4.3 (–16.7–8.2)		2.7 (–10.4–15.8)		4.6 (–6.2–15.3)	
Polypharmacy at both ages	–25.1 (–35.6 to –14.6)		–18.0 (–29.5 to –6.4)		–9.8 (–19.3 to –0.3)	
Addenbrooke's Cognitive Examination, Third Edition, n = 1529		<.001		.05		
Polypharmacy at 60–64 only	–0.3 (–1.5–0.9)		0.3 (–0.9–1.4)			
Polypharmacy at 69 only	–2.3 (–3.3 to –1.2)		–1.0 (–2.0–0.1)			
Polypharmacy at both ages	–2.4 (–3.3 to –1.5)		–1.0 (–2.0 to –0.1)			
**Physical**						
Chair rise speed, stands/min, n = 1,634		<.001		<.001		<.001
Polypharmacy at 60–64 only	–3.5 (–5.1 to –1.9)		–2.5 (–4.1 to –0.9)		–1.2 (–2.6–0.3)	
Polypharmacy at 69 only	–3.5 (–4.9 to –2.0)		–1.8 (–3.3 to –0.3)		–1.2 (–2.6–0.1)	
Polypharmacy at both ages	–6.2 (–7.4 to –5.0)		–3.9 (–5.2 to –2.6)		–2.4 (–3.6 to –1.2)	
Walking speed, m/s, n = 1,566		<.001		<.001		.007
Polypharmacy at 60–64 only	–0.1 (–0.2 to –0.1)		–0.1 (–0.1–0.0)		0.0 (–0.1–0.0)	
Polypharmacy at 69 only	–0.1 (–0.2–0.0)		0.0 (–0.1–0.0)		0.0 (–0.1–0.0)	
Polypharmacy at both ages	–0.2 (–0.3 to –0.2)		–0.1 (–0.2 to –0.1)		–0.1 (–0.1–0.0)	
Standing balance time, log seconds, n = 1,716		<.001		.02		.03
Polypharmacy at 60–64 only	–0.1 (–0.2–0.0)		0.0 (–0.2–0.1)		0.0 (–0.1–0.1)	
Polypharmacy at 69 only	–0.2 (–0.3 to –0.1)		–0.1 (–0.2–0.0)		–0.1 (–0.2–0.0)	
Polypharmacy at both ages	–0.3 (–0.4 to –0.2)		–0.1 (–0.2–0.0)		–0.1 (–0.2–0.0)	
Grip strength, kg, n = 1,644		<.001		<.001		<.001
Polypharmacy at 60–64 only	–1.6 (–2.9 to –0.2)		–0.9 (–2.2–0.4)		–0.1 (–1.2–1.1)	
Polypharmacy at 69 only	–3.1 (–4.3 to –1.8)		–2.6 (–3.8 to –1.3)		–1.6 (–2.7 to –0.5)	
Polypharmacy at both ages	–3.1 (–4.1 to –2.1)		–2.1 (–3.2 to –1.0)		–1.9 (–2.9 to –0.9)	

Polypharmacy defined as ≥5 medications.

Model 1: Adjusted for sex.

Model 2: Adjusted for sex, education and disease burden, plus body mass index (BMI) and height in models of physical capability.

Model 3: Adjusted for sex, education, disease burden, and equivalent outcome measure 60–64, plus BMI and height in models of physical capability.

Reference: no polypharmacy at either age.

a Difference in mean score.

CI = confidence interval.

Participants with polypharmacy at age 60 to 64 had slower mean chair rise speed (–2.5 stands/min, 95% CI=−4.1 to −0.9) and walking speed (–0.1 m/s, 95% CI=−0.1–0.0) at age 69 than those with no polypharmacy at either age. Effect sizes were slightly weaker when comparing those with polypharmacy at age 69 only with those with no polypharmacy at either age (chair rise speed: −1.8 stands/min, 95% CI=−3.3 to −0.3; walking speed: 0.0 m/s, 95% CI=−0.1 to 0.0), although there was no evidence that these 2 groups with polypharmacy at 60 to 64 or 69 only differed when their effect estimates were compared.

Sensitivity analyses conducted on the maximum sample did not alter these results. Participants taking no medication had better cognitive and physical capability than those prescribed 1 to 4 medications (who were combined in the reference group for the main analyses) (data provided in Supplementary Table S3), although mean capability in the group taking 1 to 4 medications was markedly better than that of those with polypharmacy.

## Discussion

In a relatively large, nationally representative study population, polypharmacy was associated with poorer cognitive and physical capability at age 69. In all cases in which there were associations, excessive polypharmacy was associated with poorer physical and cognitive capability than polypharmacy alone. Associations were stronger when there was exposure to polypharmacy on at least two occasions, as opposed to a single occasion. These findings suggest that there are dose‐dependent, cumulative negative associations between polypharmacy and cognitive and physical capability.

A major strength of NSHD is that it is an age‐homogenous birth cohort that is representative of the U.K. general population born in the post‐war era.^11^, ^12^ Continuous measures of cognitive and physical capability provide more detailed outcomes for analyses than are measurable in routine health record databases. In this sample, detailed information allowed us to control for determinants of polypharmacy, such as education and disease burden,^2^ that might confound associations with capability outcomes. Longitudinal data also allowed us to examine effects of cumulative exposure to polypharmacy. Limitations of this study include sample attrition due to participant loss to follow‐up, which is inherent to all studies of ageing populations,^20^ although there was no notable difference in the prevalence of polypharmacy between 60 to 64 and 69 or in any measure of cognitive and physical capability at age 69 when included participants were compared with participants excluded because of missing data. As such, participant loss is unlikely to have altered the pattern of associations observed. An additional limitation of this study is that medication and disease burden data relied on self‐report, albeit collected by research nurses using prescription lists and diagnostic prompts. Evidence suggests that self‐reported measures of medication correlate well with pharmacy prescription records,^21^ and omission from self‐reports of medications not taken may partly account for medication nonadherence, which is a limitation of studies using health record data to ascertain this information. The accuracy of self‐reported diagnosed disease varies according to condition severity, although reporting for most major diseases has high accuracy using this measure.^22^ Poorer baseline capability in those with excessive and repeated polypharmacy might partly explain negative associations between polypharmacy and capability, because these participants have a higher proportion of physically limiting conditions for which medication has been prescribed. To minimize this bias by indication, we adjusted for disease burden and severity and additionally for baseline measures of capability. Disease severity is included to reduce disparity between diagnosed diseases. Certain conditions, such as congestive heart failure, warrant multiple medications and may be more physically and cognitively disabling than, for example, gastroesophageal reflux disease. By additionally adjusting for long‐term limiting illness, health problem, or disability, this potential bias is partially mitigated, although it cannot be entirely eliminated. Although adjustments for disease severity and baseline capability reduced effect sizes marginally, the trend toward poorer capability in the presence of polypharmacy was maintained.

Our definition of polypharmacy is based on a numerical count of prescribed medications, which although widely accepted, is not without limitations.^17^ When translating findings to clinical practice, correct optimization of pharmacological and nonpharmacological therapies for disease management may still benefit capability. The negative changes associated with polypharmacy outlined here may be due to inappropriate or overtreatment or specific “problem medications,” such as anticholinergic therapies. Further work is required to explore this.

Our study found consistent evidence of associations between polypharmacy and cognitive capability.^3^, ^23^ In contrast, in another study, an association was found between polypharmacy and lower MMSE scores only in those taking more than 10 medications.^6^ The fact that our measures of cognitive capability were more detailed and finely graded than the MMSE may explain this difference. Although prior work on lower limb function assessed using standing balance, chair rise speed, and a timed 3‐m walk also found associations between polypharmacy and impairment,^4^ we found that these associations were stronger in individuals taking more medications. In addition to findings related to lower limb function, we observed an association with lower grip strength. This measure is a component of many commonly used measures of frailty, which prior research has found to be associated with polypharmacy in older populations.^24^ Reductions in balance observed in our study may indicate greater risk of falls, which also have strong associations with polypharmacy.^3^, ^25^, ^26^

In addition to the suggestion of a dose response for polypharmacy (with more medications associated with poorer capability), a contribution of our study is the suggestion that these associations are more pronounced in those with cumulative exposure to polypharmacy (at ages 60–64 and 69). Any increase in number of medications prescribed to an individual raises the risk of unexpected drug‐drug interactions and side effects,^1^ many of which could lead to impaired cognitive or physical capability. Additionally, individuals may become sensitized to medications or downregulate internal homeostatic or metabolic processes when experiencing prolonged exposure to a medication.^27^, ^28^ This may underlie the cumulative burden of polypharmacy observed here, with effects becoming more apparent in later life, when vulnerability to subtle impairments in cognitive and physical capability increases. One such example might be seen in the findings of a previous study^29^ that found associations between polypharmacy and incident dementia in a large Taiwanese cohort. Subtle reductions in cognitive capability related to polypharmacy may have exposed preexisting dementia or been an early expression of dementia provoked by polypharmacy. With regard to our own findings on cognition, we estimate that the regression coefficient of –2.9 for the association between excessive polypharmacy and ACE‐III score would result in the majority of those scoring just above the validated clinical threshold for potential dementia (82/100, nearly 7% of participants with ACE‐III data) to dip below this score, warranting referral for a clinical investigation. In addition, existing evidence links decline in the physical and cognitive capability measures used with mortality, independent of preexisting health status.^30^, ^31^

Further trials of medication reduction should not only assess possible subtle improvements in physical and cognitive outcomes, but should also factor in the longitudinal nature of polypharmacy. These studies should consider the possibility that reducing polypharmacy earlier in the course of its development, in addition to optimizing disease control, might avoid potential cumulative detrimental effects on physical and cognitive capability noted here in early old age.

## Supporting information


**Table S1**. Standardized Cross‐sectional Associations of Polypharmacy with Cognitive and Physical Capability at Age 69 (Data for Figure 1)
**Table S2**. Standardized Longitudinal Associations of Polypharmacy with Cognitive and Physical Capability (Data for Figure 2)
**Table S3**. Sensitivity Analyses for Cross‐sectional Associations of Polypharmacy with Cognitive and Physical Capability at Age 69, Including Additional Separation of ‘No Polypharmacy’ into ‘No Medication’ and ‘One to Four Medications’
