# Supplementary material for: Associations Between Polypharmacy and Cognitive and Physical Capability: A British Birth Cohort Study
Source: J Am Geriatr Soc. 2018 Mar 24;66(5):916–23. doi: 10.1111/jgs.15317 (PMC6001617; doi:10.1111/jgs.15317)
Supplement: Supplementary file 1 — Table S1. Standardized Cross‐sectional Associations of Polypharmacy with Cognitive and Physical Capability at Age 69 (Data for Figure 1) Table S2. Standardized Longitudinal Associations of Polypharmacy with Cognitive and Physical Capability (Data for Figure 2) Table S3. Sensitivity Analyses for Cross‐sectional Associations of Polypharmacy with Cognitive and Physical Capability at Age 69, Including Additional Separation of ‘No Polypharmacy’ into ‘No Medication’ and ‘One to Four Medications’ [file JGS-66-916-s001.docx]

**SUPPLEMENTAL DATA**

**TABLE S1: Standardised Cross-sectional Associations of Polypharmacy with Cognitive and Physical Capability at Age 69 (Data for Figure 1)**

|  |  | ***Model 2*** | | | |
| --- | --- | --- | --- | --- | --- |
| ***COGNITIVE OUTCOMES*** | | β | 95% LCI | 95% UCI | p |
| ***Word Learning Task***  *n=1934* | No Polypharmacy  Polypharmacy  Excessive Polypharmacy | (ref)  -0.10  -0.24 | -0.22  -0.45 | 0.01  -0.03 | 0.036 |
| ***Verbal Search  Speed Task***  *n=1964* | No Polypharmacy  Polypharmacy  Excessive Polypharmacy | (ref)  -0.14  -0.23 | -0.26  -0.46 | -0.01  -0.01 | 0.027 |
| ***ACE-iii***  *n=1673* | No Polypharmacy  Polypharmacy  Excessive Polypharmacy | (ref)  -0.19  -0.27 | -0.31  -0.50 | -0.07  -0.05 | 0.002 |
| ***PHYSICAL OUTCOMES*** | |  | | | |
| ***Chair Rise Speed (stands/min)***  *n=1864* | No Polypharmacy  Polypharmacy  Excessive Polypharmacy | (ref)  -0.26  -0.43 | -0.38  -0.65 | -0.14  -0.02 | <0.001 |
| ***Walking Speed (m/s)***  *n=1876* | No Polypharmacy  Polypharmacy  Excessive Polypharmacy | (ref)  -0.14  -0.51 | -0.26  -0.72 | -0.02  -0.29 | <0.001 |
| ***Standing Balance Time***  ***(Log Seconds)***  *n=1955* | No Polypharmacy  Polypharmacy  Excessive Polypharmacy | (ref)  -0.15  -0.23 | -0.27  -0.46 | -0.03  -0.01 | 0.019 |
| ***Grip Strength (kg)***  *n=1978* | No Polypharmacy  Polypharmacy  Excessive Polypharmacy | (ref)  -0.18  -0.34 | -0.26  -0.49 | -0.10  -0.20 | <0.001 |

No Polypharmacy = 0-4 Medications, Polypharmacy = 5-8 Medications, Excessive Polypharmacy = 9+ Medications
Model 2: Adjusted for Gender, Education and Disease Burden plus BMI and Height in models of physical capability.**TABLE S2: Standardised Longitudinal Associations of Polypharmacy with Cognitive and Physical Capability (Data for Figure 2)**

|  |  | ***Model 2*** | | | |
| --- | --- | --- | --- | --- | --- |
| ***COGNITIVE OUTCOMES*** | | β | 95% LCI | 95% UCI | p |
| ***Word Learning Task***  *n=1675* | No Polypharmacy at either age  Polypharmacy at 60-64 only  Polypharmacy at 69 only  Polypharmacy at both ages | (ref)  -0.08  -0.07  -0.17 | -0.26  -0.23  -0.31 | 0.09  0.08  -0.03 | 0.113 |
| ***Verbal Search  Speed Task***  *n=1705* | No Polypharmacy at either age  Polypharmacy at 60-64 only  Polypharmacy at 69 only  Polypharmacy at both ages | (ref)  0.07  0.01  -0.25 | -0.13  -0.15  -0.40 | 0.25  0.18  -0.10 | 0.005 |
| ***ACE-iii***  *n=1529* | No Polypharmacy at either age  Polypharmacy at 60-64 only  Polypharmacy at 69 only  Polypharmacy at both ages | (ref)  0.04  -0.16  -0.17 | -0.15  -0.33  -0.33 | 0.23  0.01  -0.02 | 0.053 |
| ***PHYSICAL OUTCOMES*** | |  | | | |
| ***Chair Rise Speed (stands/min)***  *n=1634* | No Polypharmacy at either age  Polypharmacy at 60-64 only  Polypharmacy at 69 only  Polypharmacy at both ages | (ref)  -0.27  -0.15  -0.44 | -0.45  -0.31  -0.59 | -0.09  0.01  -0.29 | <0.001 |
| ***Walking Speed (m/s)***  *n=1566* | No Polypharmacy at either age  Polypharmacy at 60-64 only  Polypharmacy at 69 only  Polypharmacy at both ages | (ref)  -0.17  -0.07  -0.35 | -0.35  -0.23  -0.50 | 0.01  0.08  -0.21 | <0.001 |
| ***Standing Balance Time***  ***(Log Seconds)***  *n=1716* | No Polypharmacy at either age  Polypharmacy at 60-64 only  Polypharmacy at 69 only  Polypharmacy at both ages | (ref)  -0.08  -0.16  -0.20 | -0.27  -0.32  -0.35 | 0.10  0.01  -0.05 | 0.035 |
| ***Grip Strength (kg)***  *n=1644* | No Polypharmacy at either age  Polypharmacy at 60-64 only  Polypharmacy at 69 only  Polypharmacy at both ages | (ref)  -0.10  -0.25  -0.19 | -0.22  -0.35  -0.28 | 0.02  -0.14  -0.09 | <0.001 |

Polypharmacy = 5+ Medications.
Model 2: Adjusted for Gender, Education and Disease Burden plus BMI and Height in models of physical capability.

**TABLE S3: Sensitivity Analyses for Cross-sectional Associations of Polypharmacy with Cognitive and Physical Capability at Age 69, Including Additional Separation of ‘No Polypharmacy’ into ‘No Medication’ and ‘One to Four Medications’**

|  |  | ***Model 1*** | | | | ***Model 2*** | | | |
| --- | --- | --- | --- | --- | --- | --- | --- | --- | --- |
| ***COGNITIVE OUTCOMES*** | | β | 95% LCI | 95% UCI | p | β | 95% LCI | 95% UCI | p |
| ***Word Learning Task***  *n=1934* | No Medications  One to Four Medications  Polypharmacy  Excessive Polypharmacy | (ref)  -0.9  -2.3  -3.9 | -1.6  -3.2  -5.3 | -0.2  -1.5  -2.5 | <0.001 | (ref)  -0.6  -1.1  -2.0 | -1.3  -2.0  -3.4 | 0.1  -0.2  -0.6 | 0.023 |
| ***Verbal Search  Speed Task***  *n=1964* | No Medications  One to Four Medications  Polypharmacy  Excessive Polypharmacy | (ref)  -11.5  -25.6  -36.1 | -20.0  -36.2  -52.8 | -3.0  -15.1  -19.4 | <0.001 | (ref)  -6.5  -15.7  -23.0 | -15.4  -27.5  -41.4 | 2.4  -3.8  -4.6 | 0.026 |
| ***ACE-iii***  *n=1673* | No Medications  One to Four Medications  Polypharmacy  Excessive Polypharmacy | (ref)  -0.6  -2.7  -3.9 | -1.3  -3.7  -5.4 | 0.2  -1.8  -2.4 | <0.001 | (ref)  0.0  -1.2  -1.7 | -0.8  -2.2  -3.2 | 0.7  -0.2  -0.2 | 0.007 |
| ***PHYSICAL OUTCOMES*** | |  |  |  |  |  | | | |
| ***Chair Rise Speed (stands/min)***  *n=1864* | No Medications  One to Four Medications  Polypharmacy  Excessive Polypharmacy | (ref)  -2.2  -5.8  -8.6 | -3.1  -7.0  -10.6 | -1.2  -4.5  -6.7 | <0.001 | (ref)  -1.3  -3.3  -4.9 | -2.3  -4.7  -7.0 | -0.3  -2.0  -2.8 | <0.001 |
| ***Walking Speed (m/s)***  *n=1876* | No Medications  One to Four Medications  Polypharmacy  Excessive Polypharmacy | (ref)  -0.1  -0.2  -0.4 | -0.1  -0.2  -0.4 | 0.0  -0.1  -0.3 | <0.001 | (ref)  0.0  -0.1  -0.2 | -0.1  -0.1  -0.2 | 0.0  0.0  -0.1 | <0.001 |
| ***Standing Balance Time***  ***(Log Seconds)***  *n=1955* | No Medications  One to Four Medications  Polypharmacy  Excessive Polypharmacy | (ref)  -0.1  -0.3  -0.4 | -0.2  -0.4  -0.6 | -0.1  -0.2  -0.3 | <0.001 | (ref)  -0.1  -0.1  -0.2 | -0.1  -0.2  -0.3 | 0.0  -0.1  -0.1 | 0.007 |
| ***Grip Strength (kg)***  *n=1978* | No Medications  One to Four Medications  Polypharmacy  Excessive Polypharmacy | (ref)  -1.0  -3.7  -6.1 | -1.9  -4.8  -7.8 | -0.2  -2.7  -4.5 | <0.001 | (ref)  -0.3  -2.3  -4.0 | -1.1  -3.4  -5.7 | 0.5  -1.2  -2.3 | <0.001 |

Polypharmacy = 5-8 Medications, Excessive Polypharmacy = 9+ Medications
Model 1: Gender Adjusted. Model 2: Adjusted for Gender, Education and Disease Burden plus BMI and Height in models of physical capability.
